# 4-Chloro­selanyl-3,5-diethyl-1*H*-pyrazol-2-ium chloride

**DOI:** 10.1107/S1600536811043790

**Published:** 2011-10-29

**Authors:** Maksym Seredyuk, Kateryna O. Znovjyak, Tetyana Yu. Sliva, Matti Haukka, Igor O. Fritsky

**Affiliations:** a National Taras Shevchenko University, Department of Chemistry, Volodymyrska Street 64, 01033 Kyiv, Ukraine; b Department of Chemistry, University of Joensuu, PO Box 111, 80101 Joensuu, Finland

## Abstract

In the cation of the title compound, C_7_H_12_ClN_2_Se^+^·Cl^−^, the ethyl­ene groups and the Se–Cl fragment adopt a *cis* configuration with a C—Se—Cl angle of 96.09 (6)°. In the crystal, inter­molecular N—H⋯Cl hydrogen bonds link two cations and two chlorine anions into centrosymmetric clusters. π–π inter­actions between the pyrazole rings [centroid–centroid distance of 3.530 (2) Å] link these clusters into columns along [001] with short inter­molecular Se⋯Cl^−^ contacts of 2.995 (1) Å.

## Related literature

For reviews of organoselenium chemistry, see: Krief (1995 ▶); Freudendahl *et al.* (2009 ▶). For structural studies of bis­(1*H*-pyrazol-4-yl)selenides, see: Seredyuk, Fritsky *et al.* (2010 ▶). For structural studies of *d*-metal complexes of bis­(1*H*-pyrazol-4-yl)selenides, see: Seredyuk *et al.* (2007 ▶, 2009 ▶); Seredyuk, Moroz *et al.* (2010 ▶).
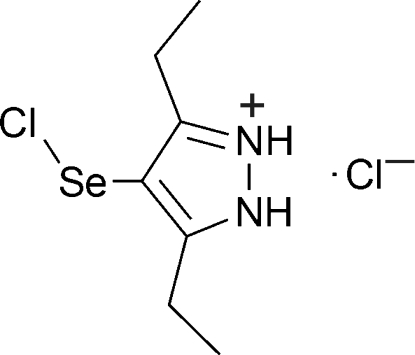


## Experimental

### 

#### Crystal data

C_7_H_12_ClN_2_Se^+^·Cl^−^
*M* *_r_* = 274.05
Monoclinic, 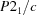

*a* = 8.1944 (6) Å
*b* = 19.3719 (10) Å
*c* = 7.1241 (3) Å
β = 111.025 (6)°
*V* = 1055.60 (10) Å^3^
*Z* = 4
Mo *K*α radiation
μ = 4.01 mm^−1^
*T* = 120 K
0.30 × 0.21 × 0.10 mm

#### Data collection

Nonius KappaCCD diffractometer
Absorption correction: multi-scan (*XPREP* in *SHELXTL*; Sheldrick, 2008 ▶) *T*_min_ = 0.379, *T*_max_ = 0.699
13385 measured reflections
2423 independent reflections
2040 reflections with *I* > 2σ(*I*)
*R*_int_ = 0.032

#### Refinement

*R*[*F* ^2^ > 2σ(*F* ^2^)] = 0.026
*wR*(*F* ^2^) = 0.049
*S* = 1.04
2423 reflections
119 parameters
H atoms treated by a mixture of independent and constrained refinement
Δρ_max_ = 0.50 e Å^−3^
Δρ_min_ = −0.38 e Å^−3^

### 

Data collection: *COLLECT* (Bruker–Nonius, 2004 ▶); cell refinement: *DENZO*/*SCALEPACK* (Otwinowski & Minor, 1997 ▶); data reduction: *DENZO*/*SCALEPACK*; program(s) used to solve structure: *SIR2004* (Burla *et al.*, 2003 ▶); program(s) used to refine structure: *SHELXL97* (Sheldrick, 2008 ▶); molecular graphics: *DIAMOND* (Brandenburg, 2005 ▶); software used to prepare material for publication: *SHELXL97*.

## Supplementary Material

Crystal structure: contains datablock(s) global, I. DOI: 10.1107/S1600536811043790/cv5179sup1.cif

Structure factors: contains datablock(s) I. DOI: 10.1107/S1600536811043790/cv5179Isup2.hkl

Supplementary material file. DOI: 10.1107/S1600536811043790/cv5179Isup3.cml

Additional supplementary materials:  crystallographic information; 3D view; checkCIF report

## Figures and Tables

**Table 1:** Hydrogen-bond geometry (Å, °)

*D*—H⋯*A*	*D*—H	H⋯*A*	*D*⋯*A*	*D*—H⋯*A*
N1—H1⋯Cl1	0.82 (3)	2.22 (3)	3.0333 (19)	172 (3)
N2—H2⋯Cl1^i^	0.81 (3)	2.22 (3)	3.030 (2)	178 (2)

Symmetry code: (i) 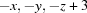.

**Fig. 1.**
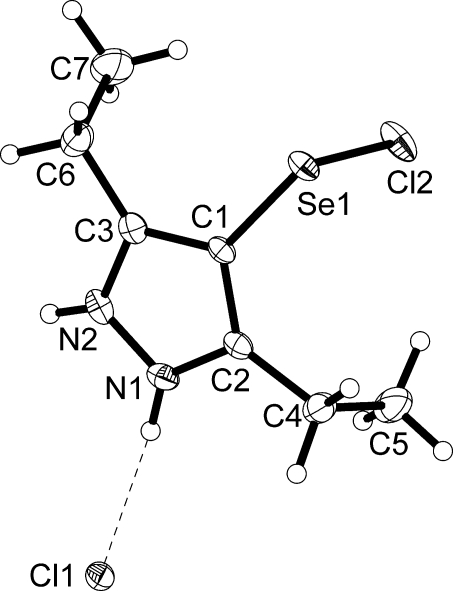
The content of asymmetric part of the title compound showing the atomic numbering and 50% probability displacement ellipsoids. Dashed line denotes hydrogen bond.

## supplementary crystallographic information

### Comment

Aryl selenides are central reagents in organoselenium chemistry (Krief, 1995; Freudendahl *et al.*, 2009). Pyrazole-based selenides are promising multidentate ligands for supramolecular frameworks and complexes of 3*d*-metals (Seredyuk *et al.*, 2007; Seredyuk *et al.*, 2009; Seredyuk, Moroz *et al.*, 2010). As a part of our study of the bis(1*H*-pyrazol-4-yl)selenides (Seredyuk, Fritsky *et al.*, 2010), we report the crystal structure of the title compound (Fig. 1).

In the title compound, between pairs of molecules of the compound strong N—H···Cl hydrogen bonds are observed with the distance N···Cl being 3.030 (2) and 3.0333 (19) Å (Table 1). The crystal packing exhibits π···π interactions between the pyrazol rings (centroid-centroid distance is 3.530 (2) Å) and short intermolecular Se···Cl^-^ contacts of 2.995 (1) Å.

### Experimental

Mixture of 3,5-diethyl-1*H*-pyrazole (1.241 g, 10 mmol), selenium dioxide (1.670 g, 15 mmol) and pyridine (25 ml) was refluxed 6 h, after that pyridine was distilled off under reduced pressure. Syrup-like residue was dissolved in 20 ml of conc. HCl and put in a fridge at 4°C for one week. The obtained precipitate was filtered off and dried. In the obtained mixture, well formed orange crystals are the target compound, whereas yellowish crystals are hydrochloride of bis(3,5-diethyl-1*H*-pyrazol-4-yl)selenide. C_7_H_12_Cl_2_N_2_Se requires: C, 30.68; H, 4.41; N, 10.22. Found: C, 30.44; H, 4.54; N, 10.20.

### Refinement

N-bound H atoms were located on a difference Fourier map and refined isotropically. Other H atoms were placed in idealized position and constrained to ride on their parent atoms with the distances 0.98–0.99 Å and with *U*_iso_ = 1.2–1.5_eq_ (parent atom).

### Crystal data

**Table:**

C_7_H_12_ClN_2_Se^+^·Cl^−^	*F*(000) = 544
*M**_r_* = 274.05	*D*_x_ = 1.724 Mg m^−^^3^
Monoclinic, *P*2_1_/*c*	Mo *K*α radiation, λ = 0.71073 Å
Hall symbol: -P 2ybc	Cell parameters from 3966 reflections
*a* = 8.1944 (6) Å	θ = 1.0–27.5°
*b* = 19.3719 (10) Å	µ = 4.01 mm^−^^1^
*c* = 7.1241 (3) Å	*T* = 120 K
β = 111.025 (6)°	Block, orange
*V* = 1055.60 (10) Å^3^	0.30 × 0.21 × 0.10 mm
*Z* = 4	

### Data collection

**Table:**

Nonius KappaCCD diffractometer	2423 independent reflections
Radiation source: fine-focus sealed tube	2040 reflections with *I* > 2σ(*I*)
horizontally mounted graphite crystal	*R*_int_ = 0.032
Detector resolution: 9 pixels mm^-1^	θ_max_ = 27.5°, θ_min_ = 2.7°
φ scans and ω scans with κ offset	*h* = −10→10
Absorption correction: multi-scan (*XPREP* in *SHELXTL*; Sheldrick, 2008)	*k* = −23→25
*T*_min_ = 0.379, *T*_max_ = 0.699	*l* = −9→9
13385 measured reflections	

### Refinement

**Table:**

Refinement on *F*^2^	Primary atom site location: structure-invariant direct methods
Least-squares matrix: full	Secondary atom site location: difference Fourier map
*R*[*F*^2^ > 2σ(*F*^2^)] = 0.026	Hydrogen site location: inferred from neighbouring sites
*wR*(*F*^2^) = 0.049	H atoms treated by a mixture of independent and constrained refinement
*S* = 1.04	*w* = 1/[σ^2^(*F*_o_^2^) + (0.0163*P*)^2^ + 1.0004*P*] where *P* = (*F*_o_^2^ + 2*F*_c_^2^)/3
2423 reflections	(Δ/σ)_max_ = 0.001
119 parameters	Δρ_max_ = 0.50 e Å^−^^3^
0 restraints	Δρ_min_ = −0.38 e Å^−^^3^

### Special details

**Table:**

Geometry. All e.s.d.'s (except the e.s.d. in the dihedral angle between two l.s. planes) are estimated using the full covariance matrix. The cell e.s.d.'s are taken into account individually in the estimation of e.s.d.'s in distances, angles and torsion angles; correlations between e.s.d.'s in cell parameters are only used when they are defined by crystal symmetry. An approximate (isotropic) treatment of cell e.s.d.'s is used for estimating e.s.d.'s involving l.s. planes.
Refinement. Refinement of *F*^2^ against ALL reflections. The weighted *R*-factor *wR* and goodness of fit *S* are based on *F*^2^, conventional *R*-factors *R* are based on *F*, with *F* set to zero for negative *F*^2^. The threshold expression of *F*^2^ > σ(*F*^2^) is used only for calculating *R*-factors(gt) *etc*. and is not relevant to the choice of reflections for refinement. *R*-factors based on *F*^2^ are statistically about twice as large as those based on *F*, and *R*- factors based on ALL data will be even larger.

### Fractional atomic coordinates and isotropic or equivalent isotropic displacement parameters (Å^2^)

**Table:**

	*x*	*y*	*z*	*U*_iso_*/*U*_eq_	
Se1	0.09775 (3)	0.154947 (11)	0.72449 (3)	0.01773 (7)	
Cl1	−0.26973 (7)	−0.02283 (3)	1.32107 (8)	0.01820 (12)	
Cl2	0.00330 (8)	0.25802 (3)	0.77369 (9)	0.02668 (14)	
N1	−0.0543 (3)	0.06371 (10)	1.1409 (3)	0.0188 (4)	
H1	−0.119 (4)	0.0437 (15)	1.188 (4)	0.040 (8)*	
N2	0.1181 (3)	0.06797 (9)	1.2431 (3)	0.0192 (4)	
H2	0.162 (3)	0.0557 (13)	1.359 (4)	0.023 (7)*	
C1	0.0658 (3)	0.11191 (10)	0.9452 (3)	0.0144 (4)	
C2	−0.0913 (3)	0.08920 (10)	0.9570 (3)	0.0158 (4)	
C3	0.1960 (3)	0.09761 (10)	1.1294 (3)	0.0164 (4)	
C4	−0.2731 (3)	0.09086 (12)	0.8111 (4)	0.0235 (5)	
H4A	−0.3384	0.0512	0.8368	0.028*	
H4B	−0.2718	0.0860	0.6733	0.028*	
C5	−0.3658 (3)	0.15699 (13)	0.8246 (5)	0.0352 (6)	
H5A	−0.3615	0.1634	0.9627	0.053*	
H5B	−0.4880	0.1545	0.7333	0.053*	
H5C	−0.3082	0.1960	0.7864	0.053*	
C6	0.3877 (3)	0.10861 (12)	1.2043 (4)	0.0247 (5)	
H6A	0.4335	0.0933	1.1001	0.030*	
H6B	0.4428	0.0796	1.3251	0.030*	
C7	0.4393 (3)	0.18281 (13)	1.2570 (4)	0.0353 (6)	
H7A	0.3884	0.2117	1.1371	0.053*	
H7B	0.5670	0.1869	1.3064	0.053*	
H7C	0.3960	0.1982	1.3617	0.053*	

### Atomic displacement parameters (Å^2^)

**Table:**

	*U*^11^	*U*^22^	*U*^33^	*U*^12^	*U*^13^	*U*^23^
Se1	0.02534 (13)	0.01435 (11)	0.01690 (11)	−0.00085 (9)	0.01169 (9)	0.00111 (9)
Cl1	0.0180 (3)	0.0205 (3)	0.0173 (3)	0.0003 (2)	0.0077 (2)	0.0031 (2)
Cl2	0.0454 (4)	0.0144 (3)	0.0276 (3)	0.0047 (2)	0.0220 (3)	0.0042 (2)
N1	0.0212 (11)	0.0159 (9)	0.0228 (10)	−0.0016 (8)	0.0122 (9)	0.0014 (8)
N2	0.0281 (11)	0.0145 (9)	0.0156 (10)	0.0028 (8)	0.0084 (9)	0.0021 (8)
C1	0.0201 (11)	0.0097 (10)	0.0153 (10)	−0.0002 (8)	0.0086 (9)	−0.0004 (8)
C2	0.0209 (12)	0.0095 (9)	0.0194 (11)	0.0004 (8)	0.0101 (10)	−0.0010 (8)
C3	0.0218 (12)	0.0097 (10)	0.0178 (11)	0.0011 (8)	0.0072 (9)	−0.0030 (8)
C4	0.0181 (12)	0.0209 (12)	0.0307 (13)	−0.0004 (9)	0.0076 (10)	−0.0010 (10)
C5	0.0208 (12)	0.0229 (13)	0.0586 (18)	0.0025 (11)	0.0103 (12)	0.0011 (12)
C6	0.0196 (12)	0.0232 (12)	0.0276 (12)	0.0002 (10)	0.0039 (10)	−0.0023 (10)
C7	0.0311 (15)	0.0263 (13)	0.0429 (16)	−0.0088 (11)	0.0063 (13)	−0.0067 (12)

### Geometric parameters (Å, °)

**Table:**

Se1—C1	1.8793 (19)	C4—H4A	0.9900
Se1—Cl2	2.2144 (6)	C4—H4B	0.9900
N1—C2	1.329 (3)	C5—H5A	0.9800
N1—N2	1.339 (3)	C5—H5B	0.9800
N1—H1	0.82 (3)	C5—H5C	0.9800
N2—C3	1.328 (3)	C6—C7	1.507 (3)
N2—H2	0.81 (3)	C6—H6A	0.9900
C1—C3	1.390 (3)	C6—H6B	0.9900
C1—C2	1.391 (3)	C7—H7A	0.9800
C2—C4	1.480 (3)	C7—H7B	0.9800
C3—C6	1.482 (3)	C7—H7C	0.9800
C4—C5	1.510 (3)		
			
C1—Se1—Cl2	96.09 (6)	C5—C4—H4B	109.2
C2—N1—N2	109.64 (18)	H4A—C4—H4B	107.9
C2—N1—H1	129 (2)	C4—C5—H5A	109.5
N2—N1—H1	121 (2)	C4—C5—H5B	109.5
C3—N2—N1	109.82 (19)	H5A—C5—H5B	109.5
C3—N2—H2	128.2 (18)	C4—C5—H5C	109.5
N1—N2—H2	121.9 (18)	H5A—C5—H5C	109.5
C3—C1—C2	107.06 (18)	H5B—C5—H5C	109.5
C3—C1—Se1	126.01 (16)	C3—C6—C7	113.1 (2)
C2—C1—Se1	126.93 (16)	C3—C6—H6A	109.0
N1—C2—C1	106.75 (19)	C7—C6—H6A	109.0
N1—C2—C4	121.10 (19)	C3—C6—H6B	109.0
C1—C2—C4	132.13 (19)	C7—C6—H6B	109.0
N2—C3—C1	106.72 (19)	H6A—C6—H6B	107.8
N2—C3—C6	121.5 (2)	C6—C7—H7A	109.5
C1—C3—C6	131.8 (2)	C6—C7—H7B	109.5
C2—C4—C5	112.1 (2)	H7A—C7—H7B	109.5
C2—C4—H4A	109.2	C6—C7—H7C	109.5
C5—C4—H4A	109.2	H7A—C7—H7C	109.5
C2—C4—H4B	109.2	H7B—C7—H7C	109.5
			
C2—N1—N2—C3	1.3 (2)	N1—N2—C3—C6	−179.63 (18)
Cl2—Se1—C1—C3	−101.57 (18)	C2—C1—C3—N2	0.0 (2)
Cl2—Se1—C1—C2	77.53 (18)	Se1—C1—C3—N2	179.22 (14)
N2—N1—C2—C1	−1.3 (2)	C2—C1—C3—C6	178.7 (2)
N2—N1—C2—C4	179.96 (18)	Se1—C1—C3—C6	−2.1 (3)
C3—C1—C2—N1	0.8 (2)	N1—C2—C4—C5	90.0 (3)
Se1—C1—C2—N1	−178.43 (15)	C1—C2—C4—C5	−88.4 (3)
C3—C1—C2—C4	179.4 (2)	N2—C3—C6—C7	−105.4 (3)
Se1—C1—C2—C4	0.1 (3)	C1—C3—C6—C7	76.0 (3)
N1—N2—C3—C1	−0.8 (2)		

### Hydrogen-bond geometry (Å, °)

**Table:**

*D*—H···*A*	*D*—H	H···*A*	*D*···*A*	*D*—H···*A*
N1—H1···Cl1	0.82 (3)	2.22 (3)	3.0333 (19)	172 (3)
N2—H2···Cl1^i^	0.81 (3)	2.22 (3)	3.030 (2)	178 (2)

Symmetry codes: (i) −*x*, −*y*, −*z*+3.
